# Validation and clinical implementation of cerebrospinal fluid C-reactive protein for the diagnosis of bacterial meningitis: a prospective diagnostic accuracy study

**DOI:** 10.1016/j.lanepe.2025.101309

**Published:** 2025-04-29

**Authors:** Sabine E. Olie, Steven L. Staal, Nina S. Groeneveld, Hans H.M. Schotman, Jacob Bodilsen, Henrik Nielsen, Merijn W. Bijlsma, Diederik van de Beek, Matthijs C. Brouwer

**Affiliations:** a Department of Neurology, Amsterdam Neuroscience, Amsterdam UMC, University of Amsterdam, Amsterdam, the Netherlands; b European Society for Clinical Microbiology and Infectious Disease (ESCMID) Study Group on Infections of the Brain (ESGIB), Basel, Switzerland; c Department of Clinical Chemistry, Amsterdam UMC, Vrije Universiteit Amsterdam, Amsterdam, the Netherlands; d Department of Infectious Diseases, Aalborg University Hospital, Aalborg, Denmark; e Department of Clinical Medicine, Aalborg University, Aalborg, Denmark; f Department of Pediatrics, Amsterdam UMC, University of Amsterdam, Amsterdam, the Netherlands

**Keywords:** C-reactive protein, Bacterial meningitis, Diagnostic accuracy, Implementation study, Cerebrospinal fluid

## Abstract

**Background:**

C-reactive protein (CRP) in cerebrospinal fluid (CSF) was previously shown to be predictive for bacterial meningitis in patients with a suspected central nervous system (CNS) infection in an experimental study. We aimed to assess the diagnostic accuracy of CRP in CSF in a validation and clinical implementation study.

**Methods:**

We validated CRP measurements in CSF for the diagnosis of bacterial meningitis in a Danish cohort of patients with acute CNS infections, and a Dutch cohort of pediatric patients suspected of a CNS infection. Subsequently, we evaluated the implementation of CRP measurements in CSF in clinical practice.

**Findings:**

CRP in CSF was measured in 103 adult patients from Denmark, which included 34 (33%) bacterial meningitis patients. The AUC was 0.92 (95% CI: 0.85–0.99), and with a predefined cut-off of 0.3 mg/L, sensitivity was 85% (95% CI: 69–95) with a specificity of 96% (95% CI: 88–99). In 77 Dutch children, including 17 (22%) patients with bacterial meningitis, the AUC was 0.95 (95% CI: 0.87–1.00) and sensitivity and specificity were 94% (95% CI: 71–100) and 98% (95% CI: 91–100), respectively. From June 2024 to November 2024, we included 80 patients in our clinical implementation cohort, of which 15 (19%) were diagnosed with bacterial meningitis. The AUC for CRP in CSF was 0.99 (95% CI: 0.97–1.00), and sensitivity was 100% (95% CI: 78–100) with a specificity of 94% (95% CI: 85–99). Across all cohorts, the combination of CSF leukocytes and CSF CRP improved diagnostic accuracy compared to CSF leukocytes alone (p ≤ 0.001 in all cohorts).

**Interpretation:**

CRP in CSF is a highly reliable predictor for bacterial meningitis, offering incremental value in addition to CSF leukocytes. Clinical implementation is straightforward and can be achieved at low costs in laboratories where CRP in blood is already routinely measured.

**Funding:**

Supported by the 10.13039/501100000781European Research Council (ERC Consolidator grant 101001237 to MB) and the 10.13039/501100001826Netherlands Organisation for Health Research and Development (ZonMw; NWO-Vidi Grant [917.17.308] to MCB; NWO-Vici-Grant Grant [918.19.627] to DvdB).

**Research in context**

**Evidence before this study**

We searched the PubMed database for studies on the use of C-reactive protein (CRP) in cerebrospinal fluid (CSF) for the diagnosis of bacterial meningitis, until September 2024. The search terms we used were ‘bacterial meningitis, ‘C-reactive protein’, ‘cerebrospinal fluid’, ‘diagnostics’, and ‘diagnostic accuracy’. A 2024 systematic review on the accuracy of CSF biomarkers for the diagnosis of pediatric meningitis found a high diagnostic accuracy for CSF CRP, with a summarised AUC of 0.94 (95% CI: 0.92–0.97) for differentiating bacterial from viral meningitis. However, results showed considerable heterogeneity and most studies included only patients with a confirmed bacterial meningitis while excluding patients with other disorders, which limits generalizability to clinical practice.

**Added value of this study**

We assessed the diagnostic accuracy of CRP in CSF for the diagnosis of bacterial meningitis in two validation cohorts, followed by a clinical implementation cohort at our institution. CRP in CSF proved to be a highly reliable marker for bacterial meningitis across all age groups in the European population, offering valuable diagnostic information in addition to CSF leukocyte count. Its easy integration into laboratories that routinely measure CRP in blood makes CRP in CSF a practical and cost-effective addition to current clinical practice.

**Implications of all the available evidence**

Our study confirmed that CSF CRP is an excellent diagnostic marker for bacterial meningitis, and that its implementation in clinical practice is simple and available at low cost. This makes it a valuable tool for diagnosing bacterial meningitis by enabling timely and accurate treatment, which is essential for a good prognosis.

## Introduction

Accurate and timely diagnosis of bacterial meningitis is crucial for improving outcome.^1^^,^^2^ Cerebrospinal fluid (CSF) examination is pivotal, with CSF leukocyte count being the most commonly used predictor.^3^^,^^4^ Yet, overlapping CSF profiles with other central nervous system (CNS) infections and non-infectious neurological disorders impact specificity and can complicate the diagnostic process.^3^^,^^5^^,^^6^ Notably, approximately 2% of bacterial meningitis cases present with a normal CSF leukocyte count.^3^^,^^7^, ^8^, ^9^ The gold standard remains detection of the causative pathogen via CSF culture or polymerase chain reaction. However, these methods are often time-intensive, and their effectiveness depends on factors such as the specific pathogen and timing of antibiotic administration.^4^^,^^10^ Therefore, there is an ongoing need for innovative approaches to facilitate the rapid and accurate identification of bacterial meningitis.

CRP is an acute-phase reactant produced in the liver and released into the bloodstream in response to systemic inflammation. Its blood concentration is commonly used as an indicator of systemic infections and to differentiate between bacterial and non-bacterial infections.^11^^,^^12^ We showed that CRP levels in CSF, measured using a Luminex assay, alongside IL-6 and IL-1β, can accurately distinguish bacterial meningitis from other disorders in a cohort of 778 patients with suspected CNS infection, regardless of CSF leukocyte count.^7^ Given that CRP measurements in blood are routinely performed in most clinical laboratories, we hypothesised that implementation of CRP measurement in CSF into clinical practice could be achieved with relative ease.

In this study we examined precision of the standard blood CRP application for CSF through repeatability and reproducibility measurements. Subsequently, we validated CRP in CSF as a diagnostic marker for bacterial meningitis in adult and pediatric patient populations. Finally, we evaluated the implementation of CRP measurements in CSF in our hospital and assessed diagnostic accuracy for the diagnosis of bacterial meningitis in routine clinical practice.

## Methods

### Stability and precision measurements

The index test of the study was the CRP concentration measured in CSF using the Roche Diagnostics (Almere, the Netherlands) CRP4 Tina-quant C-reactive Protein IV assay, according to manufacturer’s instructions. The limit of detection (LoD) of the assay was 0.30 mg/L, which we predefined to be the cut-off value for a positive test. All patients with a CRP value below the limit of detection were assigned the value 0.00 mg/L. We verified stability of CRP in CSF using CSF samples from our previous project, in which we measured multiple inflammatory markers, including CRP, using Luminex technology.^7^ Repeatability was measured between the limit of blank (LoB, 0.2 mg/L) and LoD (0.3 mg/L), measuring CRP in CSF (0.248 mg/L) ten times in a single run. Reproducibility and intermediate precision were calculated after triple measurements in two consecutive days at two levels (0.248 mg/L and 1.53 mg/L). The reliability of CRP below the level of quantification (LoQ) (0.6 mg/L) was tested during a dilution linearity test (1:1 and 1:2) using diluent solution (Roche) and a CSF sample with an unmeasurable CRP concentration.

### Danish validation cohort

For the international validation of CRP in CSF, we included patients from the DASGIB cohort (Danish Study Group of Infections of the Brain) at Aalborg University Hospital, Denmark. Methods of this study have been previously described in detail.^13^ In short, the DASGIB study is a nationwide prospective cohort study, and all adult (≥15 years) patients admitted with a community-acquired CNS infection are included. The final diagnosis of bacterial meningitis, the reference standard, was based on a combination of a clinical presentation consistent with bacterial meningitis, and detection of the causative bacterium (by CSF or blood culture, CSF polymerase chain reaction (PCR), or CSF antigen tests). In patients without microbiological confirmation, a CSF leukocyte count >10/mm^3^ was required, and the final diagnosis was made by a specialist in neurological infections. In case of uncertainty, cases were discussed with the DASGIB secretary and chair or at meetings. The study was approved by The Danish Data Protection Agency (record no. 2012-58-0018). By Danish law, studies using existing data sources do not require patient consent. For the current study we included patients from whom sufficient leftover CSF was available for CRP analysis. The laboratory technician who performed the CRP measurements was blinded to the final diagnosis.

### Pediatric validation cohort

For the validation of CRP in CSF for the diagnosis of bacterial meningitis in children, we included patients (≤16 years of age) from the I-PACE study (Improving Prognosis by using innovative methods to diAgnose Causes of Encephalitis). Methods of this study have been previously described in detail.^7^^,^^14^ In short, the I-PACE study is a multicentre prospective biobank study in which patients of all ages who underwent CSF examination for the suspicion of a CNS infection are eligible for inclusion. We obtained written informed consent from all participants or their representatives. The study adhered to Dutch privacy legislation and was approved by the biobank ethics committee of the Amsterdam UMC (number BTC AMC2014_290). Final diagnoses for all included episodes were independently determined by two physicians (SEO, SLS or NSG), with discrepancies resolved through consultation of a neurologist specialised in neurological infections (MCB). This clinical diagnosis was considered the reference standard. A final diagnosis of bacterial meningitis was made based on a combination of a clinical presentation consistent with bacterial meningitis, and detection of the causative bacterium (by CSF or blood culture, CSF PCR, or CSF antigen tests). In patients without microbiological confirmation the final diagnosis was made by consultation of a neurologist specialised in neurological infections (MCB). For the current study, we included all patients ≤16 years of age from whom sufficient leftover CSF was available for CRP analysis. The laboratory technician who performed the CRP measurements was blinded to the final diagnosis.

### Clinical implementation cohort

In June 2024, CRP measurement in CSF was introduced for routine clinical use at the Amsterdam UMC, Amsterdam, the Netherlands. CRP was measured in the acute setting and available 30–60 min after the lumbar puncture. For the current study, we utilised data from patients of all ages that were included in the I-PACE cohort in this hospital between June 2024 and November 2024.

### Statistical analysis

Statistical analyses were conducted using SPSS statistical software, version 28 (SPSS, Inc) and R Foundation for Statistical Computing, version 4.3.2. Baseline characteristics were summarised using descriptive statistics, reported as medians with interquartile ranges (IQR). Diagnostic accuracy was assessed by calculating sensitivity, specificity, positive predictive value (PPV), negative predictive value (NPV), and the area under the curve (AUC) of the receiver operator characteristics (ROC) curve, using the limit of detection of 0.3 mg/l as the predefined cut-off. To determine differences in AUC between correlated diagnostic parameters a DeLong's test was used. For the power calculation, we assumed a sensitivity of 95% based on our previous study on inflammatory markers in CSF.^7^ With an α of 0.05, marginal error (defined as the half-width of the 95% confidence interval) of 0.10 and an estimated prevalence of 10% for bacterial meningitis, the calculated sample size was 182 patients across all cohorts.^15^ This study adhered to the Standards for Reporting Diagnostic accuracy studies (STARD) checklist (Supplementary material).

### Role of funding source

The funding source has had no involvement in study design, collection analysis or interpretation of data, writing the report, or in the decision to submit the paper for publication.

## Results

### Repeatability and reproducibility

We used the CRP plasma/serum application on the Roche Cobas Pro to measure four samples from culture positive bacterial meningitis patients. With the selected samples repeatability was shown between the LoB (0.2 mg/L) and LoD (0.3 mg/L) measuring CRP in CSF (0.248 mg/L) with a mean of 0.249 mg/L, variation coefficient (VC) of 6.6% and standard deviation (SD) of 0.0164. Reproducibility (7.8% VC and 0.0214 SD) and intermediate precision (8.8% VC, 0.0242 SD) were measured at the same level as the repeatability test. Between the LoD and LoQ (0.6 mg/L) and above the LoQ, reproducibility was measured at CRP levels of 0.51 (mean 0.551, 4.7% VC, 0.0258 SD), 0.765 (mean 0.8, VC 1.4%, SD 0.0111) and 1.53 (mean 1.53, VC 2.1%, SD 0.0321) mg/L. We concluded that the performance of the analytical procedure of CRP in CSF above the LoD is reliable, reproducible, and acceptable to adjust the application for diagnostic measurements. Quantitative results are reported above 0.3 mg/L and below this threshold the results are reported as <0.3 mg/L.

### Validation and clinical implementation

We included a total of 260 patients across all cohorts, with a total of 66 (25%) bacterial meningitis cases.

### Danish validation cohort

Between May 2017 and March 2023, 336 patients were included in the DASGIB cohort, of whom 103 (31%) had sufficient CSF available for the current analysis (Supplementary Fig. S1). No significant differences were observed between patients with and without sufficient residual CSF with regard to age, sex, CSF leukocyte count, and the proportion of patients finally diagnosed with bacterial meningitis. In the 103 included Danish patients from the DASGIB cohort, 34 (33%) were diagnosed with bacterial meningitis, and 69 (67%) with a viral CNS infection. In 33 of 34 bacterial meningitis patients (97%), a relevant pathogen was identified in either blood or CSF (Supplementary Table S1). Median age was 47 years (IQR 27–70), and 49 patients (48%) were female (Table 1).

**Table 1:** Baseline characteristics of all cohorts.^a^

	Danish validation cohort^b^ (n = 103)	Pediatric validation cohort^c^ (n = 77)	Implementation cohort^d^ (n = 80)
Age (years)	47 (27–70)	3 (1–8)	45 (27–64)
Female sex	49/120 (48%)	35/77 (46%)	42/80 (53%)
**Blood chemistry**			
Blood CRP (mg/L)	11 (3–93)	24 (2–124)	18 (4–111)
Blood leukocytes (10ˆ9/L)	9.7 (6.7–13.5)	13.0 (9.4–21.4)	8.7 (6.5–15.5)
**CSF examination**			
CSF leukocytes (per mm^3^)	312 (76–1202)	4 (1–425)	7 (1–46)
CSF leukocytes ≥5/mm^3^	99/102 (97%)	33/76 (43%)	42/80 (53%)
CSF leukocytes ≥1000/mm^3^	29/102 (28%)	15/76 (20%)	8/80 (10%)
CSF protein (g/L)	0.83 (0.52–2.10)	0.33 (0.18–0.83)	0.55 (0.31–1.19)
CSF lactate (mmol/L)	3.1 (2.2–16.0)	NA	NA
CSF CRP (mg/L)	0.00 (0.00–0.41)	0.00 (0.00–0.00)	0.00 (0.00–0.00)
CSF CRP ≥ 0.30 mg/L	32/103 (31%)	17/77 (22%)	19/80 (24%)
Final diagnosis of bacterial meningitis	34/103 (33%)	17/77 (22%)	15/80 (20%)
Microbiological confirmed bacterial meningitis	33/34 (97%)	14/17 (82%)	14/15 (94%)

a Data are presented as n/N (%) or median (interquartile ranges).

b CRP in blood was known for 101 patients, leukocytes in blood for 102, CSF leukocytes for 102, CSF protein for 102, CSF lactate for 100, CSF CRP for 103 patients.

c CRP in blood was known for 68 patients, leukocytes in blood for 68, CSF leukocytes for 76, CSF protein for 77 patients, CSF CRP for 77 patients. CSF lactate was not performed.

d CRP in blood was known for 76 patients, leukocytes in blood for 78, CSF leukocytes for 80, CSF protein for 77 patients, CSF CRP for 80 patients. CSF lactate was not performed.

**Fig. 1:**
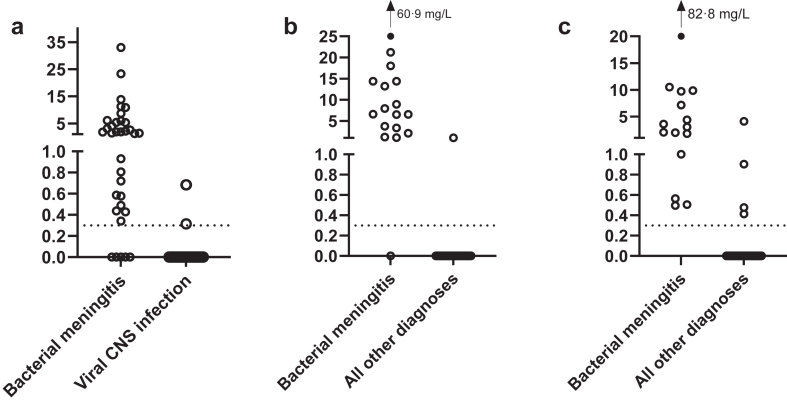
**CRP in CSF concentrations (mg/L) per cohort.** a Danish validation cohort. b Pediatric validation cohort. c Implementation cohort.

**Fig. 2:**
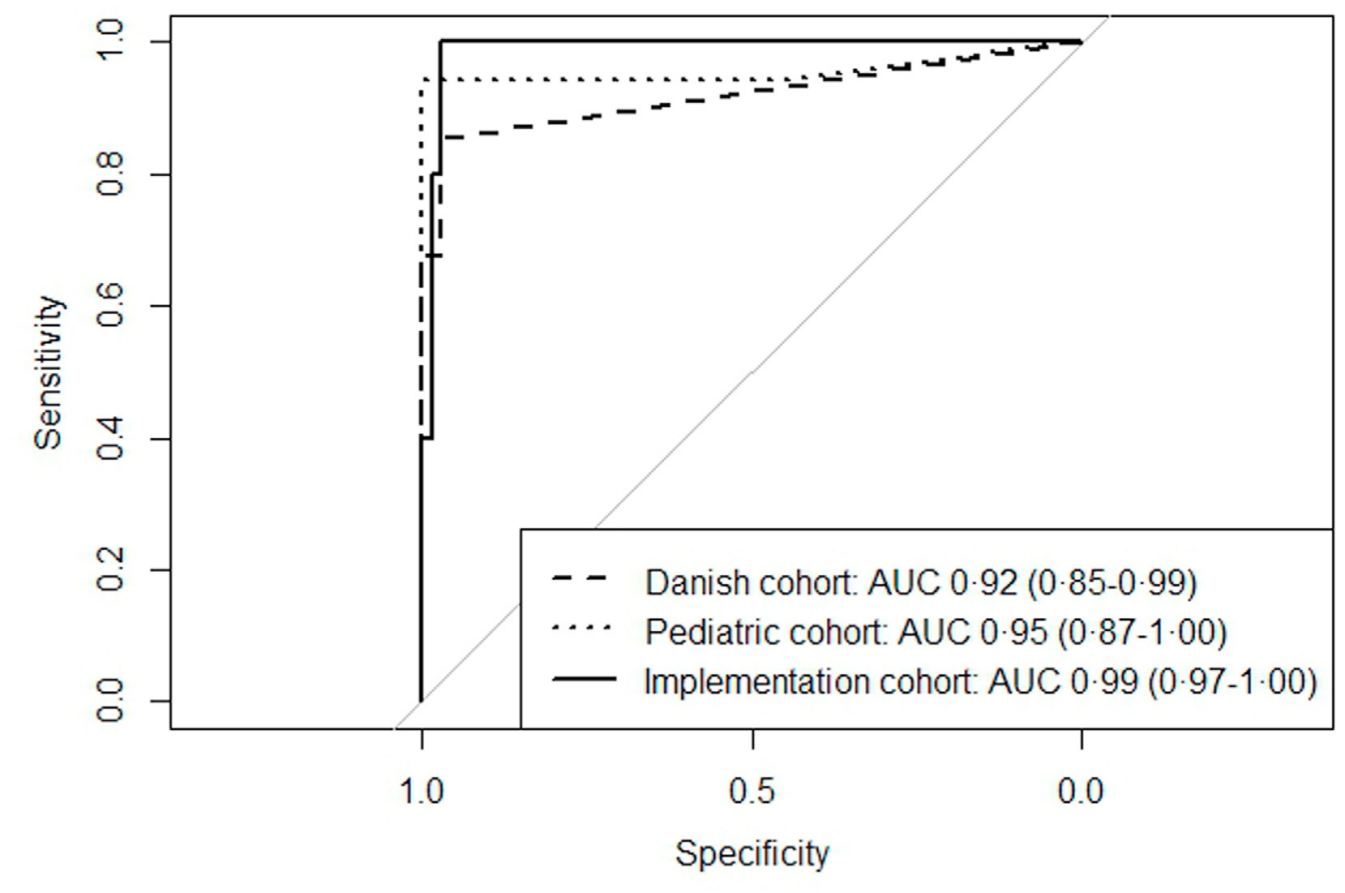
**Receiver operator curves for differentiating bacterial meningitis from other disorders**.

Median CRP concentration in CSF in the overall cohort was 0.00 (IQR 0.00–0.58), with 1.63 mg/L (IQR 0.49–5.36) in bacterial meningitis, and 0.00 mg/L (IQR 0.00–0.00) in viral CNS infections (Fig. 1, Supplementary Fig. S2 and Table S2). The AUC of CRP in CSF for differentiating bacterial meningitis from viral CNS infections was 0.92 (95% CI: 0.85–0.99; Fig. 2). While the AUC of CSF leukocytes was comparable (AUC 0.89 [95% CI: 0.82–0.96], p = 0.44), a combination of CSF CRP and CSF leukocytes outperformed CSF leukocytes alone (AUC 0.95 [0.89–1.00], p ≤ 0.001, Supplementary Table S2). Using the cut-off of 0.3 mg/L, sensitivity was 85% (95% CI: 69–95) with a specificity of 96% (95% CI: 88–99). The PPV was 91% (95% CI: 76–97) and NPV 93% (95% CI: 85–97). In five bacterial meningitis patients, CRP in CSF could not be detected, and in three patients with a viral CNS infection, CRP in CSF was elevated (Supplementary Tables S3 and S4). The correlation between CSF CRP and CRP in blood, CSF leukocytes, and CSF protein ranged from weak to moderate (r = 0.47 [95% CI: 0.31–0.61, p ≤ 0.001], r = 0.30 [95% CI: 0.11–0.47, p = 0.002] and r = 0.52 [95% CI: 0.36–0.65, p ≤ 0.001], respectively; Supplementary Table S5).

### Pediatric validation cohort

Between January 2022 and January 2024, 153 patients ≤16 years of age were included in the I-PACE study, of which 77 (50%) had sufficient CSF available for the current analysis (Supplementary Fig. S1). No significant differences were observed between patients with and without sufficient residual CSF with regard to sex, CSF leukocyte count, and the proportion of patients diagnosed with bacterial meningitis. Patients with available CSF were older than patients without available CSF (3 years [IQR 1–8] and 1 year [IQR 0–8], respectively; p = 0.02).

In the 77 included patients, a final diagnosis of bacterial meningitis was made in 17 out of 77 (22%) patients, with a relevant identified pathogen in blood or CSF in 14 (82%) cases (Supplementary Table S1). Other final diagnoses were viral CNS infections in 2 (3%) patients, CNS auto-immune disorders in 20 (26%) patients, other neurological disorders in 20 (26%) patients, and a systemic infection in 18 (23%) patients. Median age was 3 years (IQR 1–8) and 18 (23%) patients were younger than 12 months old. Of all patients 35 (46%) were female (Table 1).

The median CRP concentration in CSF in the whole cohort was 0.00 (IQR 0.00–0.00), with 6.62 mg/L (IQR 3.37–14.4) in bacterial meningitis patients, and 0.00 mg/L (IQR 0.00–0.00) in all other patients (Fig. 1, Supplementary Fig. S2 and Table S2). The AUC for differentiating bacterial meningitis from all other patients was 0.95 (95% CI: 0.87–1.00, Fig. 2). While the AUC of CSF leukocytes was similar (AUC 1.00 [95% CI: 0.99–1.00], p = 0.37), a combination of CSF CRP and CSF leukocytes outperformed CSF leukocytes alone (AUC 1.00 [1.00–1.00], p ≤ 0.001, Supplementary Table S2). Sensitivity and specificity of CSF CRP were 94% (95% CI: 71–100) and 98% (95% CI: 91–100), respectively. The PPV was 94% (95% CI: 70–99) and NPV 98% (95% CI: 90–100). In one bacterial meningitis patient CRP in CSF could not be detected (Supplementary Table S3). One patient without bacterial meningitis, with blood admixture in the CSF, had an elevated CRP concentration (Supplementary Table S4). CSF CRP showed a strong correlation with CRP in blood and CSF protein, while its correlation with CSF leukocytes was weak (r = 0.60 [95% CI: 0.42–0.73, p ≤ 0.001], r = 0.68 [95% CI: 0.54–0.79, p ≤ 0.001] and r = 0.33 [95% CI: 0.11–0.52, p = 0.004], respectively; Supplementary Table S5).

### Clinical implementation cohort

From June 2024 to November 2024, CSF CRP testing was performed in 80 patients with a suspected CNS infection in the I-PACE study. A final diagnosis of bacterial meningitis was made in 15 of 80 (19%) patients, with a relevant identified pathogen in blood or CSF in 14 (94%, Supplementary Table S1). Other diagnoses were viral CNS infection in 13 patients (16%), another CNS infection in 5 (6%), a CNS auto-immune disorder in 4 (5%), a systemic infection in 19 (24%), and a non-infectious neurological disorder in 24 (30%) patients. Median age was 45 years (IQR 27–64) and 12 (15%) patients were ≤16 years of age. Of all patients, 42 (53%) were female (Table 1).

Median CRP concentration in CSF in the whole cohort was 0.00 (IQR 0.00–0.00), with 3.02 (IQR 1.40–8.42) in bacterial meningitis patients, and 0.00 (IQR 0.00–0.00) in all other patients (Fig. 1, Supplementary Fig. S2 and Table S2). The AUC for differentiating bacterial meningitis from all other disorders was 0.99 (95% CI: 0.97–1.00), outperforming CSF leukocytes (AUC 0.89 [95% CI: 0.81–0.97], p = 0.03; Fig. 2). Combining CSF CRP with CSF leukocytes increased diagnostic accuracy, compared to CSF leukocytes alone (AUC 0.99 [0.97–1.00], p ≤ 0.001, Supplementary Table S2). For CSF CRP, using the cut-off of 0.3 mg/L, sensitivity was 100% (95% CI: 78–100) and specificity was 94% (95% CI: 85–98). The PPV was 79% (95% CI: 59–91) and NPV 100% (95% CI: 94–100). In all bacterial meningitis patients CRP in CSF was elevated, including 8 patients with a CSF leukocyte count below 1000 cells/mm^3^. Four patients with a diagnosis other than bacterial meningitis presented with an elevated CRP in the CSF (Supplementary Table S4), of whom two had blood admixture in the CSF.

The AUC of CRP in blood for differentiating bacterial meningitis from other diseases was 0.90 (0.83–0.98) and was significantly lower than CRP in CSF (p = 0.02). Additionally, the CSF to blood CRP ratio did not perform better than CRP in CSF alone (AUC 0.99 [0.97–1.00], p > 0.99). The correlation between CRP in CSF and CRP in blood was weak, between CRP in CSF and CSF leukocytes strong, and between CRP in CSF and CSF protein very strong (r = 0.38 [95% CI: 0.17–0.56, p ≤ 0.001], r = 0.74 [95% CI: 0.62–0.83, p ≤ 0.001] and r = 0.93 [95% CI: 0.88–0.95, p ≤ 0.001], respectively; Supplementary Table S5).

### Pooled results from all cohorts

The AUC of CRP in CSF for differentiating bacterial meningitis from all other patients in all cohorts combined, was 0.94 (95% CI: 0.89–0.98). While the AUC of CSF leukocytes was comparable (AUC 0.92 [95% CI: 0.87–0.96], p = 0.57), a combination of CSF CRP and CSF leukocytes outperformed CSF leukocytes alone (AUC 0.98 [0.96–1.00], p ≤ 0.001).

### CSF CRP concentration and time from treatment initiation to lumbar puncture

Across all cohorts, the timing of treatment initiation to the lumbar puncture was documented for 64 out of 66 (97%) bacterial meningitis patients. Of these patients, 32 (48%) received antibiotics prior to the lumbar puncture. For these patients, median time between initiation of treatment and the lumbar puncture was 3 h (IQR 1–9). Pearsons correlation coefficient was −0.01 (95% CI: –0.26 to 0.24, p = 0.93), indicating no association between the timing of the lumbar puncture and CSF CRP concentrations (Supplementary Fig. S3).

## Discussion

We show that CRP in CSF is a highly accurate diagnostic marker for bacterial meningitis, demonstrating 100% sensitivity and 94% specificity in a clinical implementation cohort of patients with a suspected CNS infection. CRP in CSF remains a reliable indicator even in patients with an inconclusive CSF leukocyte count (<1000/mm^3^), providing valuable diagnostic utility in clinical practice. Furthermore, administration of antibiotics prior to the lumbar puncture was not associated with lower CRP levels in patients with bacterial meningitis. Accuracy of CRP in CSF decreases in cases where blood admixture occurs during the lumbar puncture, especially in systemic infections when CRP levels in blood are high. Therefore, elevated CSF CRP concentrations in such cases should be interpreted with caution.

In our institution, we found that CRP in CSF could be seamlessly integrated into routine clinical practice, and propose that it can similarly be implemented in clinical laboratories capable of routinely measuring CRP levels in blood. CRP in CSF is a readily available and affordable diagnostic test, costing ∼ €5 per sample in the Netherlands. This could especially be an asset in low resource settings, where access to CSF cell count, Gram staining, and culture may be limited. Prospective validation of CRP in CSF in these populations is needed to confirm its accuracy.

We previously showed in the I-PACE cohort that clinical characteristics and blood parameters have a low diagnostic accuracy for the diagnosis of bacterial meningitis in a cohort of patients suspected of a CNS infection.^3^ CSF examination was found to be essential for the diagnosis with CSF leukocyte count having the highest diagnostic accuracy. However, in approximately 30% of bacterial meningitis episodes CSF leukocyte count is below 1000/mm^3^, which complicates differentiation from other CNS infections.^16^ Specifically in this population, CRP in CSF provides import additional diagnostic information which may lead to a different treatment strategy.

A 2024 systematic review on biomarkers for the diagnosis of bacterial meningitis in children found that CRP levels in CSF could correctly distinguish viral from bacterial meningitis with a pooled AUC of 0.94 (95% CI: 0.92–0.97) in 454 patients.^17^ Several other studies have previously studied CRP in CSF as a potential biomarker for bacterial meningitis, but sample sizes were often small and most studies compared culture-positive bacterial meningitis to either viral meningitis or controls with normal CSF parameters, and excluded patients with other diagnoses.^18^, ^19^, ^20^, ^21^ Clinical implementation of CRP measurement in CSF has, so far, not been described. An important strength of our implementation cohort is that we included a clinically relevant population: all patients initially suspected of a CNS infection, the population in which this test is used in clinical practice.

Our study has several limitations. First, we only included patients from the European population, which limits the global generalizability of our findings. Second, CSF lactate was not measured in the Dutch pediatric and implementation cohort, as it is not part of routine practice in the Netherlands, and as such we could not compare the diagnostic accuracy of CSF CRP with CSF lactate. However, a meta-analysis on the value of CSF lactate showed limited diagnostic accuracy in patients treated with antibiotics prior to the lumbar puncture, a substantial part of our included population.^22^ The diagnostic accuracy of CRP in CSF was not influenced by antibiotic pretreatment, suggesting a benefit over CSF lactate. Lastly, all samples used for the stability assay, and from the Danish and pediatric validation cohort underwent at least one freeze-thaw cycle, which might have affected results. Several previous studies however, have analyzed the stability of CRP in serum and found that CRP concentrations remain stable over multiple freeze-thaw cycles.^23^^,^^24^ In addition, our stability measurements showed reliable and consistent results. For the implementation study, CSF samples were used directly, and this limitation does not apply.

In conclusion, CRP in CSF is an excellent diagnostic marker for bacterial meningitis across all age groups within the European population, providing valuable diagnostic information in addition to CSF leukocyte count. Our findings were consistent in validation cohorts of Danish adults and Dutch children, and in a clinical Dutch implementation cohort of children and adults. Given it seamless integration into laboratories that routinely measure CRP in blood, CRP in CSF represents a practical and cost-effective addition to clinical practice.

## Contributors

SEO: methodology, data collection, data analysis, data interpretation, and writing of the original draft of the manuscript; SLS: data collection, review and editing of the report; NSG: data collection, review and editing of the report; HHMS: methodology, data collection, review and editing of the report; JB: data collection, review and editing of the report; HN: data collection, review and editing of the report; MWB: study design, review and editing of the report; DvdB: review, editing and supervision of the report; MCB: methodology, data interpretation, review, editing and supervision of the report, and funding acquisition. All authors read and approved the final manuscript.

SEO and MCB verified the data and had access to raw data. MCB had final responsibility for the decision to submit for publication.

## Data sharing statement

Data protection regulations in the Netherlands do not allow sharing of individual participant data. Data sets with selected aggregated data will be shared upon request. Proposals can be directed to ipace@amsterdamumc.nl.

## Declaration of interests

MWB: grant from ItsME foundation, unrelated to the current manuscript.

DvdB: grant from the Netherlands Organization for Health Research and Development, funding the current manuscript. Part of the DEX-ENCEPH trial DSMB.

MCB: grant from the Netherlands Organization for Health Research and Development and the European Research Council, funding the current manuscript. Grant from Stichting de Merel, unrelated to the current manuscript. Chair of the ESGIB study group on infections of the brain until 2024. Part of the ENCEPH-UK trial steering committee.
