# Supplementary material for: Validation and clinical implementation of cerebrospinal fluid C-reactive protein for the diagnosis of bacterial meningitis: a prospective diagnostic accuracy study
Source: Lancet Reg Health Eur. 2025 Apr 29;53:101309. doi: 10.1016/j.lanepe.2025.101309 (PMC12237792; doi:10.1016/j.lanepe.2025.101309)
Supplement: Supplementary Figs. S1–S3 and Tables S1–S5 [file mmc1.docx]

**SUPPLEMENTARY MATERIAL**

**Table of contents:**

- Supplementary Table 1 p.2

- Supplementary Table 2 p.3

- Supplementary Table 3 p.4

- Supplementary Table 4 p.5

- Supplementary Table 5 p.6

- Supplementary Figure 1 p.7

- Supplementary Figure 2 p.8

- Supplementary Figure 3 p.9

- STARD reporting guidelines p.10

**Supplementary Table 1: Bacterial meningitis pathogens in all cohorts**

| **Danish validation cohort (n= 34)** | **Pediatric validation cohort (n=17)** | **Implementation cohort (n=15)** |
| --- | --- | --- |
| *Streptococcus pneumoniae* 13 (10 CSF culture, 2 CSF PCR, 1 blood culture)  *Staphylococcus aureus* 5 (3 CSF cult, 2 blood culture)  *Haemophilus influenzae* 4 (2 CSF culture, 2 CSF PCR)  *Capnocytophaga canimorsus* 2 (2 CSF culture)  *Streptococcus anginosus* 2 (2 blood culture)  *Enterococcus faecalis* 1 (blood culture)  Group G β-hemolytic *Streptococcus* 1 (CSF culture)  Group B β-hemolytic *Streptococcus* 1 (CSF culture)  *Neisseria meningiditis* 1 (CSF culture)  *Streptococcus bovis* 1 (CSF PCR)  *Streptococcus mitis* 1 (blood culture)  *Streptococcus salivarius* 1 (CSF culture)  Culture/PCR negative 1 | *Neisseria meningiditis* 4 (3 CSF PCR, 1 CSF culture)  *Streptococcus pneumoniae* 3 (2 blood culture, 1 PCR)  *Streptococcus pyogenes* 3 (2 blood culture, 1 pus culture, 1 CSF culture)  *Haemophilus influenzae* 2 (1 blood culture, 1 CSF PCR)  *Fusobacterium necrophorum* 1 (CSF PCR)  *Salmonella* spp 1 (CSF culture)  Culture/PCR negative 3 | *Streptococcus pneumoniae* 8 (4 CSF culture, 1 CSF PCR, 3 blood culture)  *Ehrlichia* *chaffenees* 1 (specific antibodies)  *Escherichia coli* 1 (CSF culture)  *Enterococcus faecium* 1 (CSF culture)  *Fusobacterium necrophorum* 1 (blood culture)  *Staphylococcus aureus* 1 (CSF culture)  *Staphylococcus warneri* 1 (CSF and blood culture)  Culture/PCR negative 1 |

**Supplementary Table 2: Test characteristics of CSF leukocyte count using cutoffs of 5, 100, and 1000 cells/mm^3^**

|  | **Danish validation cohort** | **Pediatric validation cohort** | **Implementation cohort** |
| --- | --- | --- | --- |
| **5 cells/mm^3^** | Sens 100% (90-100)  Spec 4% (1-12)  PPV 34% (25-45)  NPV 100% (29-100) | Sens 100% (80-100)  Spec 71% (58-82)  PPV 50% (32-66)  NPV 100% (92-100) | Sens 93% (68-100)  Spec 57% (44-69)  PPV 33% (20-50)  NPV 97% (86-100) |
| **100 cells/mm^3^** | Sens 97% (85-100)  Spec 43% (31-55)  PPV 46% (34-58)  NPV 97% (83-99) | Sens 100% (80-100)  Spec 95% (86-99)  PPV 85% (62-97)  NPV 100% (94-100) | Sens 60% (32-84)  Spec 91% (81-97)  PPV 60% (32-84)  NPV 91% (81-97) |
| **1000 cells/mm^3^** | Sens 71% (53-85)  Spec 93% (84-98)  PPV 83% (64-94)  NPV 86% (76-93) | Sens 88% (64-98)  Spec 100% (94-100)  PPV 100% (78-100)  NPV 97% (87-100) | Sens 47% (21-73)  Spec 98% (91-100)  PPV 89% (47-100)  NPV 89% (79-95) |

Sens – Sensitivity, Spec – Spec, PPV – positive predictive value, NPV. – negative predictive value

**Supplementary Table 3: Bacterial meningitis cases with undetectable CRP in CSF**

| **Danish validation cohort** | **Pediatric validation cohort** | **Implementation cohort** |
| --- | --- | --- |
| *Patient 1*: Final diagnosis of *S. pneumoniae* meningitis (CSF culture). CRP in blood 15 mg/L, CRP in CSF 0·00 mg/L. CSF erythrocyte count 0/mm^3^. CSF leukocyte count 40900/mm^3^  *Patient 2*: Final diagnosis of *S. pneumoniae* meningitis (blood culture). CRP in blood 3 mg/L, CRP in CSF 0·00 mg/L. CSF erythrocyte count 0/mm^3^. CSF leukocyte count 295/mm^3^  *Patient 3*: Final diagnosis of Group G β-hemolytic Streptococcus meningitis (CSF culture). CRP in blood 218 mg/L, CRP in CSF 0·00 mg/L. CSF erythrocyte count 64/mm^3^. CSF leukocyte count 24/mm^3^  *Patient 4*: Final diagnosis of *S. mitis* meningitis (blood culture). CRP in blood 17 mg/L, CRP in CSF 0·00 mg/L. CSF erythrocyte count 0/mm^3^. CSF leukocyte count 237/mm^3^  *Patient 5*: Final diagnosis of culture-negative bacterial meningitis. CRP in blood 151 mg/L, CRP in CSF 0·00 mg/L. CSF erythrocyte count 0/mm^3^. CSF leukocyte count 295/mm^3^ | *Patient 1*: Final diagnosis of *N. meningiditis* meningitis (CSF PCR). CRP in blood 90 mg/L, CRP in CSF 0·00 mg/L. CSF erythrocyte count 1600/mm^3^. CSF leukocyte count 20834/mm^3^ | *Not applicable* |

**Supplementary Table 4: Cases without bacterial meningitis with elevated CRP in CSF**

| **Danish validation cohort** | **Pediatric validation cohort** | **Implementation cohort** |
| --- | --- | --- |
| *Patient 1*: Final diagnosis of Varicella zoster virus encephalitis. CRP in blood 40 mg/L, CRP in CSF 0·68 mg/L. CSF erythrocyte count 40/mm^3^. CSF leukocyte count 529/mm^3^.  *Patient 2*: Final diagnosis of enterovirus meningitis. CRP in blood 13 mg/L, CRP in CSF 0·32 mg/L. CSF erythrocyte count 0/mm^3^. CSF leukocyte count 332/mm^3^  *Patient 3*: Final diagnosis of Varicella zoster virus encephalitis. CRP in blood 33 mg/L, CRP in CSF 0·68 mg/L. CSF erythrocyte count 0/mm^3^. CSF leukocyte count 659/mm^3^ | *Patient 1*: Final diagnosis of systemic infection. CRP in blood 281 mg/L, CRP in CSF 1·01 mg/L. CSF erythrocyte count 2600/mm^3^. CSF leukocyte count 32/mm^3^ | *Patient 1*: Final diagnosis of sepsis. CRP in blood 206 mg/L, CRP in CSF 0·48 mg/L. CSF erythrocyte count 0/mm^3^. CSF leukocyte count 1/mm^3^  *Patient 2*: Final diagnosis of sepsis. CRP in blood 240 mg/L, CRP in CSF 4·01 mg/L. CSF erythrocyte count 0/mm^3^. CSF leukocyte count 2/mm^3^  *Patient 3*: Final diagnosis of status epilepticus due to infection. CRP in blood 160 mg/L, CRP in CSF 0·90 mg/L. CSF erythrocyte count 1600/mm^3^. CSF leukocyte count 3/mm^3^  *Patient 4*: Final diagnosis of viral meningitis. CRP in blood 41 mg/L, CRP in CSF 0·41 mg/L. CSF erythrocyte count 700/mm^3^. CSF leukocyte count 33/mm^3^ |

**Supplementary Table 5: Correlation of CRP in CSF with CRP in blood, CSF leukocytes and CSF protein**

|  | **Danish validation cohort** | **Pediatric validation cohort** | **Implementation cohort** |
| --- | --- | --- | --- |
| Pearson correlation  CRP CSF:CRP blood | 0·47 (95% CI 0·31-0·61, p =<0·001) | 0·60 (95% CI 0·42-0·73, p =<0·001) | 0·38 (95% CI 0·17-0·56, p =<0·001) |
| Pearson correlation  CRP CSF:CSF leukocytes | 0·30 (95% CI 0·11-0·47, p =0·002) | 0·33 (95% CI 0·11-0·52, p =0·004) | 0·74 (95% CI 0·62-0·83, p =<0·001) |
| Pearson correlation  CRP CSF:CSF protein | 0·52 (95% CI 0·36-0·65, p =<0·001) | 0·68 (95% CI 0·54-0·79, p =<0·001) | 0·93 (95% CI 0·88-0·95, p =<0·001) |

**Supplementary Figure 1: Flowchart of included patients**

**
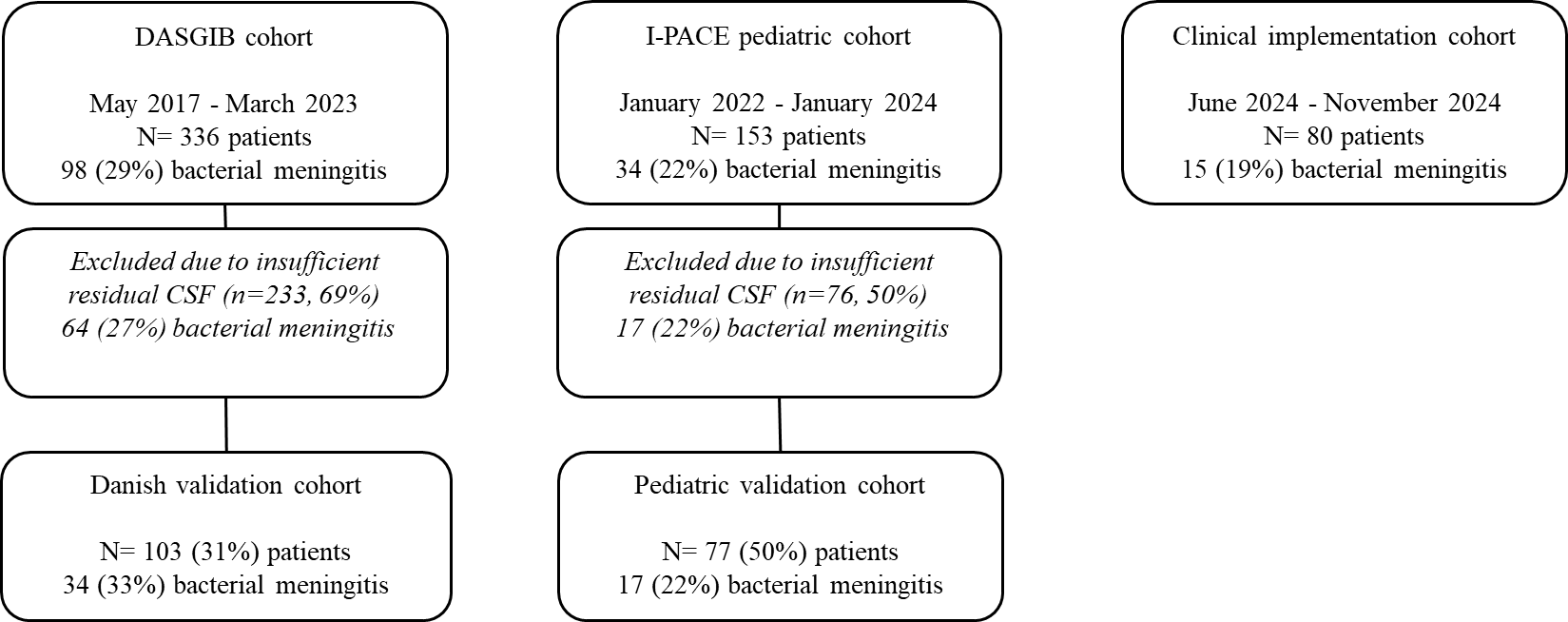
**

**Supplementary Figure 2:** **CRP in CSF concentrations (mg/L) on a logarithmic scale per cohort**


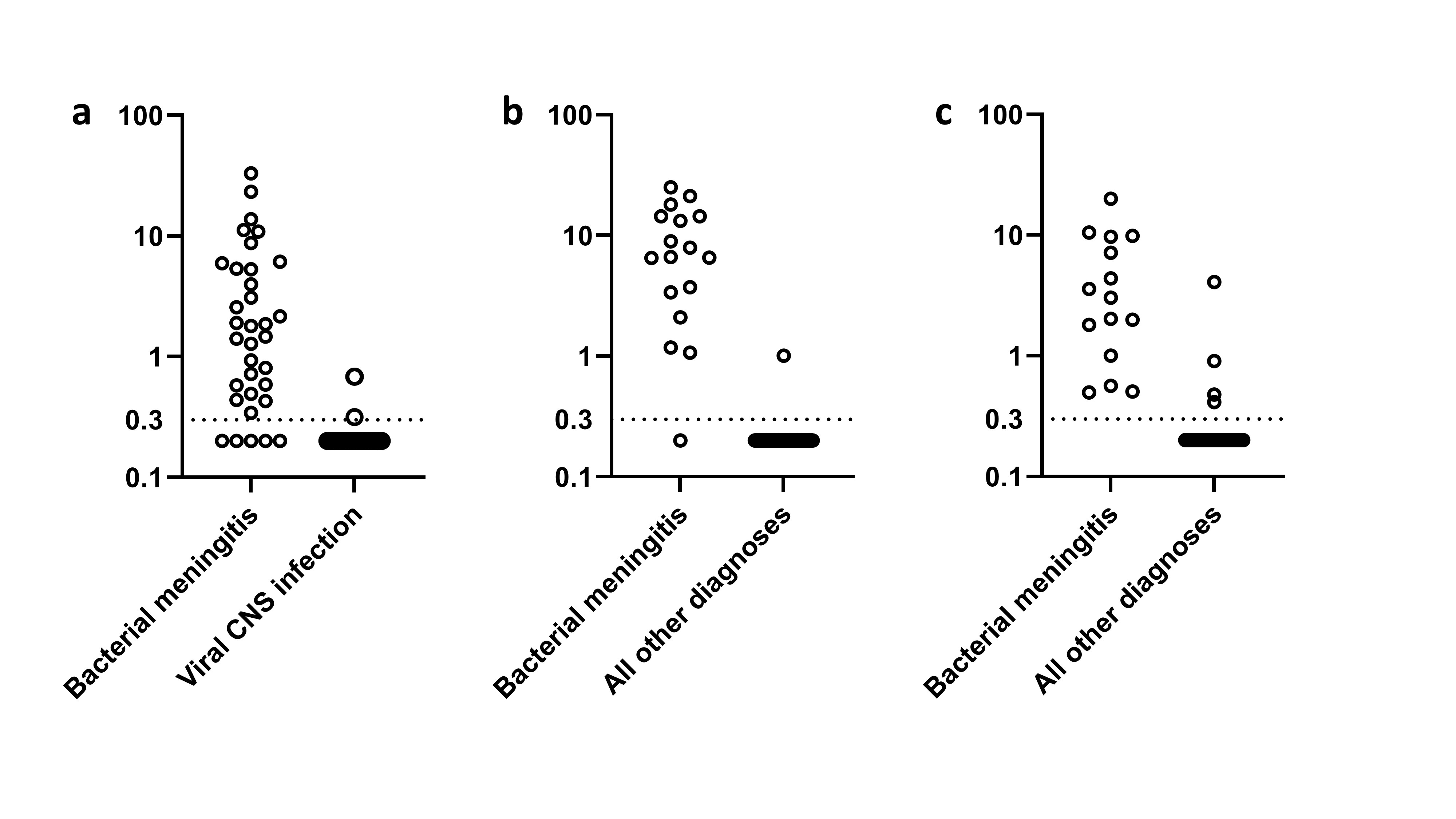


1a Danish validation cohort

1b Pediatric validation cohort

1c Implementation cohort

**Supplementary Figure 3: Correlation between CRP concentration and time from treatment initiation to lumbar puncture in bacterial meningitis cases**


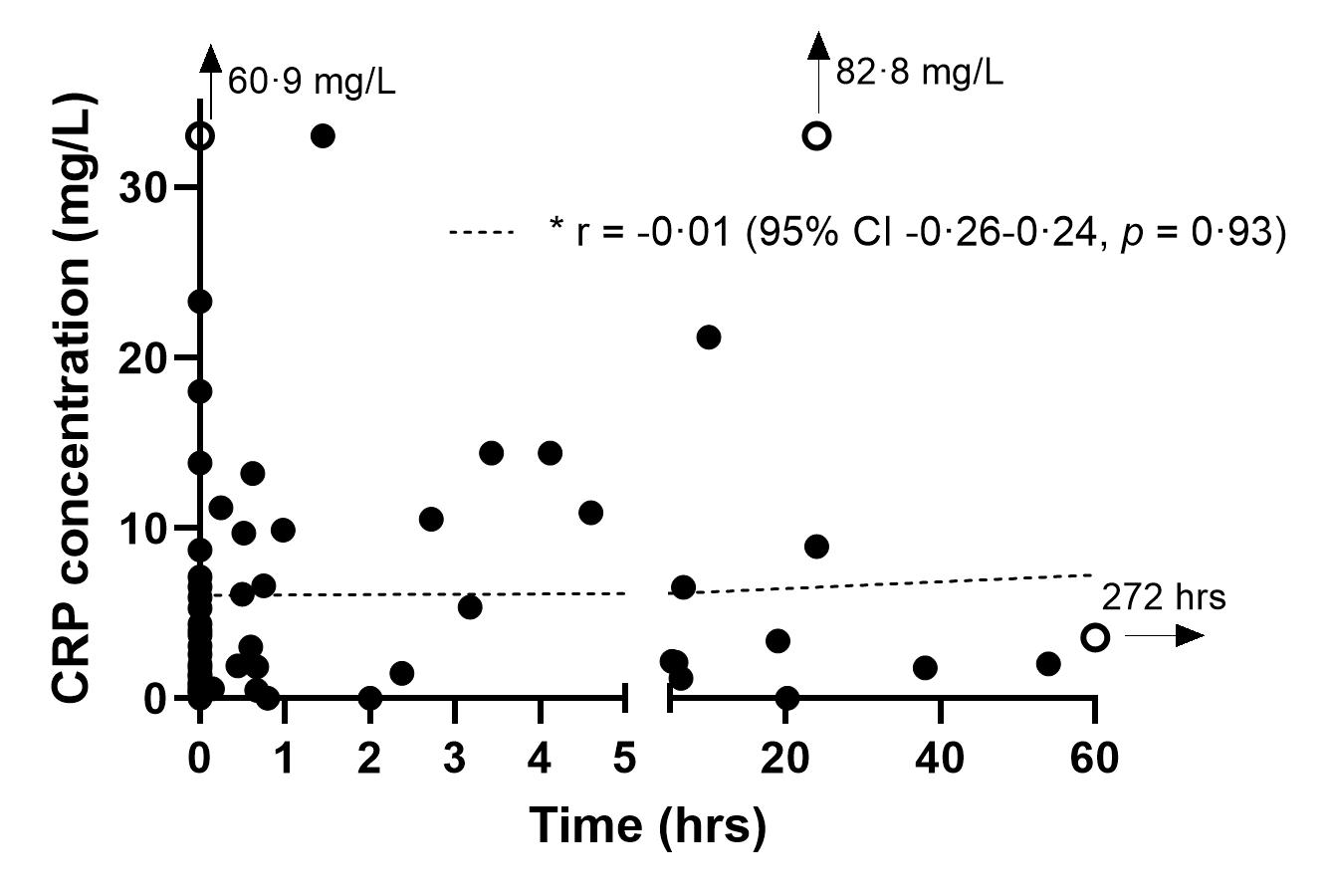


**STARD reporting guidelines**

|  | **Section & Topic** | **No** | **Item** | **Reported on page #** |
| --- | --- | --- | --- | --- |
|  |  |  |  |  |
|  | **TITLE OR ABSTRACT** |  |  |  |
|  |  | **1** | Identification as a study of diagnostic accuracy using at least one measure of accuracy  (such as Sensitivity, Specificity, predictive values, or AUC) | 2 |
|  | **ABSTRACT** |  |  |  |
|  |  | **2** | Structured summary of study design, methods, results, and conclusions  (for specific guidance, see STARD for Abstracts) | 2 |
|  | **INTRODUCTION** |  |  |  |
|  |  | **3** | Scientific and clinical background, including the intended use and clinical role of the index test | 3 |
|  |  | **4** | Study objectives and hypotheses | 3 |
|  | **METHODS** |  |  |  |
|  | *Study design* | **5** | Whether data collection was planned before the index test and reference standard  were performed (prospective study) or after (retrospective study) | 4, 5 |
|  | *Participants* | **6** | Eligibility criteria | 4, 5 |
|  |  | **7** | On what basis potentially eligible participants were identified  (such as symptoms, results from previous tests, inclusion in registry) | 4, 5 |
|  |  | **8** | Where and when potentially eligible participants were identified (setting, location and dates) | 4, 5 |
|  |  | **9** | Whether participants formed a consecutive, random or convenience series | 4, 5 |
|  | *Test methods* | **10a** | Index test, in sufficient detail to allow replication | 4 |
|  |  | **10b** | Reference standard, in sufficient detail to allow replication | 4, 5 |
|  |  | **11** | Rationale for choosing the reference standard (if alternatives exist) | 4, 5 |
|  |  | **12a** | Definition of and rationale for test positivity cut-offs or result categories  of the index test, distinguishing pre-specified from exploratory | 4 |
|  |  | **12b** | Definition of and rationale for test positivity cut-offs or result categories  of the reference standard, distinguishing pre-specified from exploratory | 4, 5 |
|  |  | **13a** | Whether clinical information and reference standard results were available  to the performers/readers of the index test | NA |
|  |  | **13b** | Whether clinical information and index test results were available  to the assessors of the reference standard | 4, 5 |
|  | *Analysis* | **14** | Methods for estimating or comparing measures of diagnostic accuracy | 5 |
|  |  | **15** | How indeterminate index test or reference standard results were handled | 4 |
|  |  | **16** | How missing data on the index test and reference standard were handled | NA |
|  |  | **17** | Any analyses of variability in diagnostic accuracy, distinguishing pre-specified from exploratory | 5 |
|  |  | **18** | Intended sample size and how it was determined | 5 |
|  | **RESULTS** |  |  |  |
|  | *Participants* | **19** | Flow of participants, using a diagram | NA |
|  |  | **20** | Baseline demographic and clinical characteristics of participants | 6, 7, 8, Table 1, Figure 1 |
|  |  | **21a** | Distribution of severity of disease in those with the target condition | NA |
|  |  | **21b** | Distribution of alternative diagnoses in those without the target condition | 6, 7, 8 |
|  |  | **22** | Time interval and any clinical interventions between index test and reference standard | NA |
|  | *Test results* | **23** | Cross tabulation of the index test results (or their distribution)  by the results of the reference standard | 6, 7, 8 |
|  |  | **24** | Estimates of diagnostic accuracy and their precision (such as 95% confidence intervals) | 6, 7, 8, Figure 2 |
|  |  | **25** | Any adverse events from performing the index test or the reference standard | NA |
|  | **DISCUSSION** |  |  |  |
|  |  | **26** | Study limitations, including sources of potential bias, statistical uncertainty, and generalisability | 10 |
|  |  | **27** | Implications for practice, including the intended use and clinical role of the index test | 9, 10 |
|  | **OTHER INFORMATION** |  |  |  |
|  |  | **28** | Registration number and name of registry | NA |
|  |  | **29** | Where the full study protocol can be accessed | NA |
|  |  | **30** | Sources of funding and other support; role of funders | 11 |
|  |  |  |  |  |
